# Hull prospective analysis of Botulinum Toxin type A (Botox) use in the treatment of chronic migraine

**DOI:** 10.1186/1129-2377-14-S1-P187

**Published:** 2013-02-21

**Authors:** F Ahmed, M Khalil, V Quarshie

**Affiliations:** 1 Hull and East Yorkshire Hospitals NHS Trust, UK

## Introduction

Botulinum toxin type A (BOTOX) is licensed for the prophylaxis of headaches in adults with chronic migraine (CM). A prospective study was performed to examine the change in frequency of CM symptoms before and after treatment with BOTOX in the real-life setting.

## Methods

Adults with CM were offered BOTOX after discussion of treatment options. Patients were injected intramuscularly as per PREEMPT, and maintained a headache diary for 30 days before/after BOTOX treatment. Data were collected for the number of headache, migraine and crystal clear (headache free) days. A responder was defined as 50% reduction in headache or migraine days, or an increment in crystal clear days twice that of the baseline in a 30-day period.

## Results

Full data were available on 67 patients (16 males (mean age 47.2 years; range 26-76 years); 51 females (mean age 42.4 years, range 19-70 years) who received BOTOX. 57/67 (85%) tried 3 preventive treatments and 36/67 (53.7%) were overusing analgesics. The median number of headache days reduced from 27 before BOTOX to 18 after BOTOX(p<0.001); the median number of migraine days reduced from 12 before to 7 after (p<0.001); the median number of crystal clear days increased from 3 before to 12 after (p<0.001). Of the cohort, 34% reported 50% reduction in headache days, 48% a 50% reduction in migraine days and 54% a 50% reduction in crystal clear days. Triptan days reduced from 8 before BOTOX to 3 after (p<0.001). Data on days off work was available for 17/67 patients; in these, the median number of days off work per month reduced from 6 before to 3 after BOTOX(0.004). 13/67(19.4%) reported adverse events; 8 with pain at injection sites, 1 with worsening headache, 3 could not frown and 1 fainted during Rx.

## Conclusions

BOTOX is a valuable addition to preventive treatment options in patients with CM. It significantly reduces the number of headache and migraine days, and significantly increases the number of crystal clear days in a real-life setting.

## Conflict of interests

FA received honorarium as advisory board member from Allergan.
